# Genotype Score for Iron Status Is Associated with Muscle Fiber Composition in Women

**DOI:** 10.3390/genes13010005

**Published:** 2021-12-21

**Authors:** Mizuki Takaragawa, Takuro Tobina, Keisuke Shiose, Ryo Kakigi, Takamasa Tsuzuki, Noriko Ichinoseki-Sekine, Hiroshi Kumagai, Hirofumi Zempo, Eri Miyamoto-Mikami, Hiroyuki Kobayashi, Hisashi Naito, Noriyuki Fuku

**Affiliations:** 1 Graduate School of Health and Sports Science, Juntendo University, Chiba 270-1695, Japan; mizuki854@gmail.com (M.T.); noriko.sekine@ouj.ac.jp (N.I.-S.); kumazin7@gmail.com (H.K.); miyamoto.mikami@gmail.com (E.M.-M.); hrkoba1@gmail.com (H.K.); hnaitou@juntendo.ac.jp (H.N.); 2 Faculty of Nursing and Nutrition, University of Nagasaki, Nagasaki 851-2195, Japan; tobitaku@sun.ac.jp; 3 Faculty of Education, University of Miyazaki, Miyazaki 889-2192, Japan; kshiose@cc.miyazaki-u.ac.jp; 4 Faculty of Management & Information Science, Josai International University, Chiba 283-8555, Japan; rkakigi@jiu.ac.jp; 5 Faculty of Pharmacy, Meijo University, Aichi 468-8503, Japan; takamasa425@gmail.com; 6 Faculty of Liberal Arts, The Open University of Japan, Chiba 261-8586, Japan; 7 Faculty of Health and Nutrition, Tokyo Seiei College, Tokyo 124-8530, Japan; zempo.hirofumi@gmail.com; 8 Mito Medical Center, Tsukuba University Hospital, Ibaraki 310-0015, Japan

**Keywords:** genetic polymorphism, transmembrane serine protease 6, *TMPRSS6*, matriptase 2, homeostatic iron regulator protein, *HFE*, anemia, iron deficiency, myosin heavy chain, skeletal muscle

## Abstract

Human muscle fiber composition is heterogeneous and mainly determined by genetic factors. A previous study reported that experimentally induced iron deficiency in rats increases the proportion of fast-twitch muscle fibers. Iron status has been reported to be affected by genetic factors. As the *TMPRSS6* rs855791 T/C and *HFE* rs1799945 C/G polymorphisms are strongly associated with iron status in humans, we hypothesized that the genotype score (GS) based on these polymorphisms could be associated with the muscle fiber composition in humans. Herein, we examined 214 Japanese individuals, comprising of 107 men and 107 women, for possible associations of the GS for iron status with the proportion of myosin heavy chain (MHC) isoforms (I, IIa, and IIx) as markers of muscle fiber composition. No statistically significant correlations were found between the GS for iron status and the proportion of MHC isoforms in all participants. When the participants were stratified based on sex, women showed positive and negative correlations of the GS with MHC-IIa (age-adjusted *p* = 0.020) and MHC-IIx (age-adjusted *p* = 0.011), respectively. In contrast, no correlation was found in men. In women, a 1-point increase in the GS was associated with 2.42% higher MHC-IIa level and 2.72% lower MHC-IIx level. Our results suggest that the GS based on the *TMPRSS6* rs855791 T/C and *HFE* rs1799945 C/G polymorphisms for iron status is associated with muscle fiber composition in women.

## 1. Introduction

The human skeletal muscle is composed of type I (slow-twitch) and type II (fast-twitch) muscle fibers. Type II muscle fibers are further divided into type IIa and IIb/x [1,2]. Type I muscle fibers contain high levels of mitochondrial oxidative phosphorylation enzymes and low levels of glycolytic enzymes, whereas those of type IIb/x contain high levels of glycolytic enzymes and low levels of mitochondrial oxidative phosphorylation enzymes. In addition, type IIa muscle fibers have properties that are an intermediate of those of type I and type IIb/x [3,4]. Type I muscle fibers specialize in endurance activities, such as long-distance running, whereas those of type IIx specialize in quick and powerful movements, such as sprinting and weightlifting. The human skeletal muscle is an extremely heterogeneous tissue composed of different muscle fiber types. A previous study has reported that large differences in muscle fiber composition (i.e., 15−85% type I fibers, 5−77% type IIa, and 0−44% type IIb) of the vastus lateralis muscle exist in the individuals of a general population [5]. This variation in muscle fiber composition explains the marked differences in physical performance among individuals [6,7]. In addition, muscle fiber composition has been associated with lifestyle-related diseases. For example, a high proportion of type I muscle fibers is associated with a lowered risk of obesity [8,9], type 2 diabetes mellitus [10], and hypertension [11]. Therefore, it is important to understand the factors that determine muscle fiber composition, which can be used for talent identification in athletes to predict the future capacity of individuals or defining the optimal training regimen for individuals, as well as for preventing metabolic diseases.

Heritability estimates of muscle fiber composition have been found to range from 45 to 99.5% [12,13]. Previous studies have reported several genetic polymorphisms associated with muscle fiber composition, such as R577X (rs1815739) in the α-actinin-3 gene (*ACTN3*) [14,15,16], I/D (rs4341) in the angiotensin-converting enzyme gene (*ACE*) [15,16], C/T (rs11549465) in the hypoxia-inducible factor 1 α gene (*HIF1A*) [17], Q472H (rs1870377) in the vascular endothelial growth factor receptor 2 gene (*VEGFR2*) [18], C/A (rs11091046) in the angiotensin II receptor, type 2 gene (*AGTR2*) [19], G482S (rs8132678) in the peroxisome proliferator-activated receptor γ coactivator-1α gene (*PPARGC1A*) [20], A/G (rs6949152) in the nuclear respiratory factor 1 gene (*NRF1*) [20], G/C (rs3213537) in the copine V gene (*CPNE5*) [17], and A/C (rs9558685) in the transforming growth factor α gene (*TGF-α*) [18]. Nevertheless, even the combined effects of genotypes for these polymorphisms are associated with only a small proportion of the variation in muscle fiber composition. Therefore, a large number of additional genetic factors that influence muscle fiber composition still need to be identified.

Iron status in the body has been suggested to be one of the factors that determines muscle fiber composition. Previous studies have reported that the muscle fiber composition in mice shifts from slow- to fast-twitch fibers when they are fed a low-iron diet [19,20]. In addition, since iron is an essential component of oxygen-binding proteins, such as hemoglobin, low iron levels limit oxygen bioavailability in the peripheral tissues, including skeletal muscle [21]. Several previous studies have reported that chronic hypoxia induces a muscle fiber composition shift from slow- to fast-twitch fibers in rats [22,23,24,25]. In humans, chronic hypoxia, such as that associated with chronic obstructive pulmonary disease (COPD) has been shown to increase the proportion of type II muscle fibers in affected individuals compared with healthy controls [26]. Taken together, these results suggest that low iron status and hypoxia, which is occasionally induced by low iron status, have been implicated in an increase in the proportion of fast-twitch muscle fibers. Accordingly, another previous study has reported that a certain polymorphism in *HIF1A*, a gene that is activated by chronic hypoxic conditions, was associated with muscle fiber composition [27]. However, the direct association between the iron status and muscle fiber composition in humans has not yet been examined.

The iron status is influenced by both genetic and environmental factors. Heritability estimates of iron status, quantified using parameters, such as serum iron, ferritin, and saturated transferrin levels, range from 23% to 29%. Recent studies have reported three genetic polymorphisms (rs855791 T/C in the transmembrane serine protease 6 gene (*TMPRSS6*), rs1800562 G/A, and rs1799945 C/G in the homeostatic iron regulator gene (*HFE*)) to be strongly associated with the iron status of an individual [28,29,30,31,32,33,34,35]. However, rs1800562 G/A in *HFE* was observed to be in a nonpolymorphic site in the genome of approximately 8300 Japanese individuals according to the Japanese Multi Omics Reference Panel (https://jmorp.megabank.tohoku.ac.jp/202001/variants, accessed on 10 October 2021). Therefore, in the present study, we focused on the two other polymorphisms, the rs855791 T/C polymorphism in *TMPRSS6* and the rs1799945 C/G polymorphism in *HFE*. We hypothesized that the genotype score (GS) based on these polymorphisms for iron status was associated with muscle fiber type composition.

## 2. Materials and Methods

### 2.1. Participants

The present study included 214 Japanese participants who were recruited from Juntendo University (30 men and 20 women, aged 27.6 ± 13.2 years) and Fukuoka University (77 men and 88 women, aged 55.1 ± 11.6 years). All participants provided their signed informed consent before enrollment in this study. This study was approved by the Ethics Committees of Juntendo University and Fukuoka University and performed in accordance with the Declaration of Helsinki.

### 2.2. Genotyping

Total DNA was isolated from the venous blood of each participant using the QIAamp DNA Blood Mini Kit (Qiagen, Hilden, Germany), according to the manufacturer’s instructions. The total DNA concentration was measured by the use of a NanoDrop 8000 spectrophotometer (Thermo Fisher Scientific, Waltham, MA, USA). Then, the DNA samples were adjusted to 10 ng/µL with a Tris-ethylenediamine tetraacetic acid (EDTA) buffer and stored at 4 °C. *TMPRSS6* rs855791 T/C and *HFE* rs1799945 C/G polymorphisms were genotyped by the use of a real-time thermocycler (QuantStudio™ 5, Applied Biosystems, Waltham, MA, USA) with TaqMan SNP Genotyping Assays (Assay ID for *TMPRSS6* rs855791: C___3289902_10, for *HFE* rs1799945: C___1085600_10). The genotyping mixture (4 µL), containing 2.5 µL of TaqMan GTXpress Master Mix (2×), 0.0625 μL of TaqMan SNP Genotyping Assay (40×), and 1.4375 μL of distilled water, was mixed with 1 μL of the total DNA (10 ng/μL) for each reaction. Four negative controls were included in each plate. Thermal cycling conditions included an initial denaturation at 95 °C for 20 s, followed by 40 cycles of denaturation at 95 °C for 3 s, and annealing/extension at 60 °C for 20 s. This thermal cycling condition included another step of cooling down to room temperature (25 °C). Subsequently, a GS based on the two studied polymorphisms for iron status was calculated using an additive effect model (i.e., 2, 1, and 0), since previous studies had reported that both the polymorphisms were associated with iron status under an additive genetic model [32]. Briefly, we assigned a score of 2 to the “high iron status” genotype (CC for *TMPRSS6* rs855791 T/C and GG for *HFE* rs1799945 C/G), a score of 1 to the “intermediate iron status” genotype (TC for *TMPRSS6* rs855791 T/C and CG for *HFE* rs1799945 C/G), and a score of 0 to the “low iron status” genotype (TT for *TMPRSS6* rs855791 T/C and CC for *HFE* rs1799945 C/G). Then, we summed the genotype scores for the two polymorphisms.

### 2.3. Myosin Heavy Chain Isoforms

Skeletal muscle samples were obtained from the vastus lateralis muscles of participants under sterile conditions and local anesthesia (1% lidocaine) using a disposable needle biopsy instrument (Max Core or Magnum; C. R. Bard, Covington, GA, USA). We conducted biopsies under ultrasound imaging (Noblus; Aloka, Tokyo, Japan) to collect tissue samples from approximately 15 cm above the lateral epicondyle of both or either leg of each participant and avoided the inclusion of subcutaneous fat and the subfascial and myotendinous parts as far as possible. In addition, any visible non-muscle tissue (e.g., fat tissue) was removed from the biopsy samples. Then, the samples were immediately frozen in liquid nitrogen and stored at −80 °C until further analysis. Myosin heavy chain (MHC) protein isoforms (I, IIa, and IIx) were analyzed by the use of sodium dodecyl-sulfate polyacrylamide gel electrophoresis (SDS-PAGE) as markers of muscle fiber composition, according to a previously described method [36].

### 2.4. Statistical Analysis

Data are expressed as the mean ± standard deviation (SD). The Hardy-Weinberg equilibrium was tested for the *TMPRSS6* rs855791 T/C and *HFE* rs1799945 C/G polymorphisms using the chi-square test. To examine the association of the GS based on the *TMPRSS6* rs855791 T/C and *HFE* rs1799945 C/G polymorphisms for iron status with muscle fiber composition, we performed linear regression analyses. Statistical significance was set at *p* < 0.05. Statistical analyses were performed using JMP Pro version 12 (SAS Institute, Cary, NC, USA).

## 3. Results

The characteristics of the participants are shown in Table 1. The men and women included in the present study did not show any significant difference among themselves with respect to age and body mass index (BMI). However, the women had a significantly lower height and body mass than those of the men (*p* < 0.001). Additionally, the proportion of MHC-I was significantly lower in men than in women (40.7 ± 11.5% vs. 50.3 ± 11.2%, *p* < 0.001), whereas the proportion of MHC-IIa (35.9 ± 8.2% vs. 30.9 ± 8.2%, *p* < 0.001) and MHC-IIx (23.4 ± 9.1% vs. 18.8 ± 8.3%, *p* = 0.001) was significantly higher in men than in women.

The genotyping success ratio was 214/214 (100%) for the *TMPRSS6* rs855791 T/C and *HFE* rs1799945 C/G polymorphisms. Neither polymorphism deviated significantly from the Hardy-Weinberg equilibrium (*p* = 0.967 and *p* = 0.700, respectively). Allele (*p* = 0.922 and 0.359) and genotype (*p* = 0.919 and 0.353) frequencies of the *TMPRSS6* rs855791 T/C and *HFE* rs1799945 C/G polymorphisms were similar between men and women. Descriptive genotypic data for the *TMPRSS6* rs855791 T/C and *HFE* rs1799945 C/G polymorphisms on the proportions of MHC-I, MHC-IIa, and MHC-IIx isoforms for all the participants are summarized in Supplemental Table S1.

**Figure 1:**
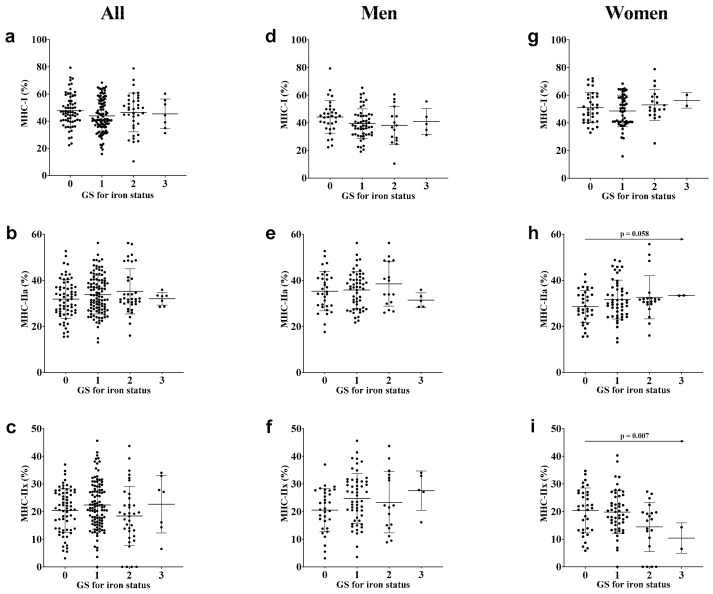
Correlations of the GS based on the *TMPRSS6* rs855791 T/C and *HFE* rs1799945 C/G polymorphisms for iron status with the proportions of MHC-I (**a**), MHC-IIa (**b**), and MHC-IIx (**c**) isoforms for all; proportions of MHC-I (**d**), MHC-IIa (**e**), and MHC-IIx (**f**) isoforms in men; and proportions of MHC-I (**g**), MHC-IIa (**h**), and MHC-IIx (**i**) isoforms in women. GS: Genotype score; MHC: Myosin heavy chain.

Data on the association of the GS based on *TMPRSS6* rs855791 T/C and *HFE* rs1799945 C/G polymorphisms for iron status with proportions of MHC-I, MHC-IIa, and MHC-IIx isoforms are presented in Figure 1 and Table 2. No statistically significant correlations were found between the GS and proportions of MHC isoforms in all participants. However, when the participants were stratified based on sex, women showed a significant negative correlation between the GS for iron status and proportion of MHC-IIx (R^2^ = 0.068, *p* = 0.007; Figure 1i), and a tendentious positive correlation between the GS and proportion of MHC-IIa (R^2^ = 0.034, *p* = 0.058; Figure 1h). After adjusting for age as a covariate, significant correlations were found between the GS and both MHC-IIa (*p* = 0.020) and MHC-IIx (*p* = 0.011) proportions. Moreover, an increase of 1 point in the GS for iron status in women was associated with a 2.42% increase in MHC-IIa level (model 2, age-adjusted *p* = 0.020) and a 2.72% decrease in MHC-IIx level (model 2, age-adjusted *p* = 0.011), as shown in Table 2. No correlation was found between the GS and the proportion of MHC-I isoform. In men, there were no statistically significant correlations in the proportions of MHC isoforms.

## 4. Discussion

The purpose of the present study was to understand the association between the GS based on the *TMPRSS6* rs855791 T/C and the *HFE* rs1799945 C/G polymorphisms for iron status and the proportions of MHC isoforms as an indicator of muscle fiber composition in 214 healthy individuals (107 men and 107 women). The results of the present study revealed that the GS for iron status was associated with muscle fiber composition in women. Particularly, the low GS for iron status (*TMPRSS6* rs855791 TT and *HFE* rs1799945 CC) was associated with a lower proportion of MHC-IIa and a higher proportion of MHC-IIx, and vice versa. To the best of our knowledge, this is the first study to examine the association between iron status-related genetic polymorphisms and muscle fiber composition in humans.

The effects of iron status on muscle fiber composition have been previously examined in rodents. It has been shown that iron deficiency anemia occurring owing to a lack of iron from food induced the transformation of muscle fibers from slow to fast-twitch fibers [19,20]. These findings suggested that iron status may also influence the muscle fiber composition in humans. Several genome-wide association studies (GWAS) have reported that the rs855791 T/C polymorphism in *TMPRSS6* and rs1799945 C/G and rs1800562 G/A polymorphisms in *HFE* were strongly associated with the iron status (i.e., serum iron, ferritin, transferrin saturation, and transferrin) of an individual [29,31,32,34,37,38,39] and explain 3.8% of the inter-individual variance in serum iron concentration [32]. Since the *HFE* rs1800562 G/A polymorphism has only been observed in European populations, we excluded it from our study as it does not contribute to the iron status in the Japanese population.

Tightly regulating the iron status in the body is critical to human health. In humans, there are no physiological pathways for iron excretion. Therefore, iron homeostasis in the body is mainly controlled by iron absorption in the small intestine, which is negatively regulated by hepcidin [40,41,42]. Hepcidin expression is mainly regulated by the hemojuvelin (HJV)/bone morphogenetic protein (BMP)/SMAD pathway, which is regulated by the products of *TMPRSS6* and *HFE* [41]. Transmembrane protease, serine 6, also known as Matriptase 2, encoded by *TMPRSS6*, suppresses hepcidin production by inhibiting the HJV/BMP/SMAD pathway when the iron levels in the blood decrease [28,40,41]. In contrast, the human homeostatic iron regulator protein, encoded by *HFE,* promotes hepcidin production by activating the HJV/BMP/SMAD pathway when the iron concentration in the blood increases [40,41]. Therefore, certain functional genetic polymorphisms in *TMPRSS6* and *HFE* might have a large impact on the iron status of the body and that might influence muscle fiber composition in humans. Indeed, in the present study, strong contributions of the GS based on the *TMPRSS6* rs855791 T/C and the *HFE* rs1799945 C/G polymorphisms for iron status to MHC-IIa (age-adjusted R^2^ = 8.7%) and MHC-IIx (age-adjusted R^2^ = 7.3%) levels were found in women. Therefore, it is suggested that the GS for iron status is one of the important factors in determining the muscle fiber composition in humans.

The specific mechanisms by which iron status-related genetic polymorphisms influence muscle fiber composition remain unclear. A major role of iron in blood is to form hemoglobin in red blood cells and to carry oxygen to peripheral tissues. In addition, iron plays essential functions in the mitochondria, which are crucial for regulating energy metabolism in the skeletal muscle. It has been speculated that iron status-related genetic polymorphisms influence muscle fiber composition by changing the mitochondrial function. Peroxisome proliferator-activated receptor γ coactivator 1α (PGC-1α) is an inducible transcription coactivator that is related to the shift in muscle fiber type via its ability to promote the mitochondrial function [43]. *PGC-1α* knockout and overexpression mice in skeletal muscle have been reported to be associated with an increased proportion of type II [44] and type I [45] muscle fibers, respectively. In line with these results, we previously reported that a G482S polymorphism (rs8132678) in the gene encoding PGC-1α (*PPARGC1A*) was associated with muscle fiber composition in humans [46]. Although the direct association between the iron status and PGC-1α activity is unclear, several animal and cell culture studies indicate that the low iron status might be one of the factors that cause a decrease in PGC-1α activity. In rats, the low iron status induces an increase in the levels of glycolytic enzymes [47] and a decrease in the levels of enzymes involved in mitochondrial oxidative phosphorylation [48], which collectively is known as the Warburg effect. It has also been reported that the removal of iron from myoblast C2C12 cells attenuates the increase in transcripts related to mitochondrial oxidative phosphorylation induced by PGC-1α overexpression [49]. As a result of this metabolic change, the activity of PGC-1α is downregulated owing to a decrease in AMP-activated protein kinase (AMPK) [50]. Therefore, the iron status-related *TMPRSS6* rs855791 T/C and *HFE* rs1799945 C/G polymorphisms may be associated with muscle fiber composition via the mitochondrial function. In the present study, significant associations between the GS for iron status and proportions of MHC isoforms were found for MHC-IIa and MHC-IIx, but not MHC-I. Although the muscle fiber shift between type IIa and IIx occurs relatively easily owing to environmental factors, such as exercise, the shift between type I and II is limited in humans [51,52]. Generally, the effect size of common genetic polymorphisms is not strong. Therefore, it is likely that the *TMPRSS6* rs855791 T/C and the *HFE* rs1799945 C/G polymorphisms do not have a large impact on the determination of the proportions of MHC-I and MHC-II. Further follow-up studies are necessary to investigate the underlying mechanism by which iron status-related genetic polymorphisms influence muscle fiber composition.

The present study has several limitations. First, the muscle fiber composition was assessed by the proportion of MHC isoforms (MHC-I, MHC-IIa, and MHC-IIx), and the number or cross-sectional area of each fiber was not measured, even though a strong correlation (R^2^ = 0.988) between the proportion of MHC-IIa as determined by SDS-PAGE and the proportion of type IIa fibers as determined by histochemical staining was found in a previous study [53]. Second, the number of participants was small. Although 214 participants were included, the sample size was relatively reduced when the participants were stratified according to sex. This could also lead to sex differences in the associations between proportions of MHC isoforms and the GS for iron status in the present study. Finally, we could not assess the iron status in the skeletal muscle as well as in the blood. However, since the *TMPRSS6* rs855791 T/C and the *HFE* rs1799945 C/G polymorphisms are strongly associated with serum iron levels, they might also influence the iron status in tissues, such as the skeletal muscle.

In summary, we investigated the associations between the iron status-related genetic polymorphisms and muscle fiber composition in Japanese individuals. Our study revealed that the GS based on the *TMPRSS6* rs855791 T/C and *HFE* rs1799945 polymorphisms for iron status was associated with muscle fiber composition in women but not in men, i.e., the low GS for iron status was associated with the low MHC-IIa and high MHC-IIx fiber proportions in the skeletal muscle of women.

## Figures and Tables

**Table 1:** Characteristics of the participants enrolled in this study.

	All (*n* = 214)	Men (*n* = 107)	Women (*n* = 107)
Age (years)	47.9 ± 17.0	47.5 ± 17.9	48.3 ± 16.3
Height (cm)	163.2 ± 8.8	169.6 ± 5.9	156.7 ± 5.9 ***
Body mass (kg)	68.9 ± 12.2	74.7 ± 11.2	62.5 ± 9.9 ***
BMI (kg/m^2^)	25.7 ± 4.0	26.0 ± 3.8	25.5 ± 4.2
MHC-I (%)	45.5 ± 12.3	40.7 ± 11.5	50.3 ± 11.2 ***
MHC-IIa (%)	33.4 ± 8.6	35.9 ± 8.2	30.9 ± 8.2 ***
MHC-IIx (%)	21.1 ± 9.0	23.4 ± 9.1	18.8 ± 8.3 ***

BMI: Body mass index; MHC: Myosin heavy chain. Data are expressed as means ± SD. *** *p* < 0.001 vs. men.

**Table 2:** Associations of the GS for iron status with the proportions of MHC-I, MHC-IIa, and MHC-IIx isoforms in women.

MHC Isoform	Model	R^2^	β for GS (95% CI), *p* Value
MHC-I	1	0.003	0.87 (−1.98–3.72), *p* = 0.546
	2	0.052	0.30 (−2.53–3.14), *p* = 0.834
MHC-IIa	1	0.034	1.99 (−0.07–4.04), *p* = 0.058
	2	0.087	2.42 (0.38–4.46), *p* = 0.020
MHC-IIx	1	0.068	−2.86 (−4.91–(−0.81)), *p* = 0.007
	2	0.073	−2.72 (−4.81–(−0.64)), *p* = 0.011

Model 1: Linear regression analysis on the GS; Model 2: Multiple linear regression analysis on the GS adjusted by age; GS: Genotype score; MHC: Myosin heavy chain; CI: Confidence interval.

## Data Availability

Not applicable.

## Supplementary Materials

The following are available online at https://www.mdpi.com/article/10.3390/genes13010005/s1. Table S1: Proportions of MHC-I, MHC-IIa, and MHC-IIx isoforms by genotypes in the *TMPRSS6* rs855791 T/C and *HFE* rs1799945 C/G polymorphis for all participants, men, and women.

## References

[1] Brooke M.H., Kaiser K.K. (1970). Muscle fiber types: How many and what kind?. Arch. Neurol..

[2] Murach K.A., Dungan C.M., Kosmac K., Voigt T.B., Tourville T.W., Miller M.S., Bamman M.M., Peterson C.A., Toth M.J. (2019). Fiber typing human skeletal muscle with fluorescent immunohistochemistry. J. Appl. Physiol..

[3] Essen B., Jansson E., Henriksson J., Taylor A.W., Saltin B. (1975). Metabolic characteristics of fibre types in human skeletal muscle. Acta Physiol. Scand..

[4] Widrick J.J., Stelzer J.E., Shoepe T.C., Garner D.P. (2002). Functional properties of human muscle fibers after short-term resistance exercise training. Am. J. Physiol. Regul Integr. Comp. Physiol..

[5] Simoneau J.A., Bouchard C. (1989). Human variation in skeletal muscle fiber-type proportion and enzyme activities. Am. J. Physiol..

[6] Zawadowska B., Majerczak J., Semik D., Karasinski J., Kolodziejski L., Kilarski W.M., Duda K., Zoladz J.A. (2004). Characteristics of myosin profile in human vastus lateralis muscle in relation to training background. Folia Histochem. Cytobiol..

[7] Ricoy J.R., Encinas A.R., Cabello A., Madero S., Arenas J. (1998). Histochemical study of the vastus lateralis muscle fibre types of athletes. J. Physiol. Biochem..

[8] Tanner C.J., Barakat H.A., Dohm G.L., Pories W.J., MacDonald K.G., Cunningham P.R., Swanson M.S., Houmard J.A. (2002). Muscle fiber type is associated with obesity and weight loss. Am. J. Physiol. Endocrinol. Metab..

[9] Gerrits M.F., Ghosh S., Kavaslar N., Hill B., Tour A., Seifert E.L., Beauchamp B., Gorman S., Stuart J., Dent R. (2010). Distinct skeletal muscle fiber characteristics and gene expression in diet-sensitive versus diet-resistant obesity. J. Lipid Res..

[10] Zierath J.R., Hawley J.A. (2004). Skeletal muscle fiber type: Influence on contractile and metabolic properties. PLoS Biol..

[11] Hernelahti M., Tikkanen H.O., Karjalainen J., Kujala U.M. (2005). Muscle fiber-type distribution as a predictor of blood pressure: A 19-year follow-up study. Hypertension.

[12] Komi P.V., Viitasalo J.H., Havu M., Thorstensson A., Sjodin B., Karlsson J. (1977). Skeletal muscle fibres and muscle enzyme activities in monozygous and dizygous twins of both sexes. Acta Physiol. Scand..

[13] Simoneau J.A., Bouchard C. (1995). Genetic determinism of fiber type proportion in human skeletal muscle. FASEB J..

[14] Vincent B., De Bock K., Ramaekers M., Van den Eede E., Van Leemputte M., Hespel P., Thomis M.A. (2007). ACTN3 (R577X) genotype is associated with fiber type distribution. Physiol. Genom..

[15] Ahmetov I.I., Druzhevskaya A.M., Lyubaeva E.V., Popov D.V., Vinogradova O.L., Williams A.G. (2011). The dependence of preferred competitive racing distance on muscle fibre type composition and ACTN3 genotype in speed skaters. Exp. Physiol..

[16] Kumagai H., Tobina T., Ichinoseki-Sekine N., Kakigi R., Tsuzuki T., Zempo H., Shiose K., Yoshimura E., Kumahara H., Ayabe M. (2018). Role of selected polymorphisms in determining muscle fiber composition in Japanese men and women. J. Appl. Physiol..

[17] Guilherme J., Semenova E.A., Zempo H., Martins G.L., Lancha Junior A.H., Miyamoto-Mikami E., Kumagai H., Tobina T., Shiose K., Kakigi R. (2020). Are Genome-Wide Association Study Identified Single-Nucleotide Polymorphisms Associated With Sprint Athletic Status? A Replication Study With 3 Different Cohorts. Int. J. Sports Physiol. Perform..

[18] Willems S.M., Wright D.J., Day F.R., Trajanoska K., Joshi P.K., Morris J.A., Matteini A.M., Garton F.C., Grarup N., Oskolkov N. (2017). Large-scale GWAS identifies multiple loci for hand grip strength providing biological insights into muscular fitness. Nat. Commun..

[19] Esteva S., Panisello P., Casas M., Torrella J.R., Pages T., Viscor G. (2008). Morphofunctional responses to anaemia in rat skeletal muscle. J. Anat..

[20] Ohira Y., Gill S.L. (1983). Effects of dietary iron deficiency on muscle fiber characteristics and whole-body distribution of hemoglobin in mice. J. Nutr..

[21] Jolly E.C., Di Boscio V., Aguirre L., Luna C.M., Berensztein S., Gene R.J. (2001). Effects of supplemental oxygen during activity in patients with advanced COPD without severe resting hypoxemia. Chest.

[22] Ishihara A., Itoh K., Itoh M., Hirofuji C. (2000). Effect of hypobaric hypoxia on rat soleus muscle fibers and their innervating motoneurons: A review. Jpn. J. Physiol..

[23] Bigard A.X., Sanchez H., Birot O., Serrurier B. (2000). Myosin heavy chain composition of skeletal muscles in young rats growing under hypobaric hypoxia conditions. J. Appl. Physiol..

[24] Faucher M., Guillot C., Marqueste T., Kipson N., Mayet-Sornay M.H., Desplanches D., Jammes Y., Badier M. (2005). Matched adaptations of electrophysiological, physiological, and histological properties of skeletal muscles in response to chronic hypoxia. Pflugers Arch..

[25] Ishihara A., Itho K., Itoh M., Hirofuji C., Hayashi H. (1994). Hypobaric-hypoxic exposure and histochemical responses of soleus muscle fibers in the rat. Acta Histochem..

[26] Gosker H.R., Zeegers M.P., Wouters E.F., Schols A.M. (2007). Muscle fibre type shifting in the vastus lateralis of patients with COPD is associated with disease severity: A systematic review and meta-analysis. Thorax.

[27] Ahmetov I.I., Hakimullina A.M., Lyubaeva E.V., Vinogradova O.L., Rogozkin V.A. (2008). Effect of HIF1A gene polymorphism on human muscle performance. Bull. Exp. Biol. Med..

[28] Nai A., Pagani A., Silvestri L., Campostrini N., Corbella M., Girelli D., Traglia M., Toniolo D., Camaschella C. (2011). TMPRSS6 rs855791 modulates hepcidin transcription in vitro and serum hepcidin levels in normal individuals. Blood.

[29] Benyamin B., Ferreira M.A., Willemsen G., Gordon S., Middelberg R.P., McEvoy B.P., Hottenga J.J., Henders A.K., Campbell M.J., Wallace L. (2009). Common variants in TMPRSS6 are associated with iron status and erythrocyte volume. Nat. Genet..

[30] Chambers J.C., Zhang W., Li Y., Sehmi J., Wass M.N., Zabaneh D., Hoggart C., Bayele H., McCarthy M.I., Peltonen L. (2009). Genome-wide association study identifies variants in TMPRSS6 associated with hemoglobin levels. Nat. Genet..

[31] Tanaka T., Roy C.N., Yao W., Matteini A., Semba R.D., Arking D., Walston J.D., Fried L.P., Singleton A., Guralnik J. (2010). A genome-wide association analysis of serum iron concentrations. Blood.

[32] Gill D., Benyamin B., Moore L.S.P., Monori G., Zhou A., Koskeridis F., Evangelou E., Laffan M., Walker A.P., Tsilidis K.K. (2019). Associations of genetically determined iron status across the phenome: A mendelian randomization study. PLoS Med..

[33] Traglia M., Girelli D., Biino G., Campostrini N., Corbella M., Sala C., Masciullo C., Vigano F., Buetti I., Pistis G. (2011). Association of HFE and TMPRSS6 genetic variants with iron and erythrocyte parameters is only in part dependent on serum hepcidin concentrations. J. Med. Genet..

[34] Kamatani Y., Matsuda K., Okada Y., Kubo M., Hosono N., Daigo Y., Nakamura Y., Kamatani N. (2010). Genome-wide association study of hematological and biochemical traits in a Japanese population. Nat. Genet..

[35] Benyamin B., Esko T., Ried J.S., Radhakrishnan A., Vermeulen S.H., Traglia M., Gogele M., Anderson D., Broer L., Podmore C. (2014). Novel loci affecting iron homeostasis and their effects in individuals at risk for hemochromatosis. Nat. Commun..

[36] Kakigi R., Naito H., Ogura Y., Kobayashi H., Saga N., Ichinoseki-Sekine N., Yoshihara T., Katamoto S. (2011). Heat stress enhances mTOR signaling after resistance exercise in human skeletal muscle. J. Physiol. Sci..

[37] Benyamin B., McRae A.F., Zhu G., Gordon S., Henders A.K., Palotie A., Peltonen L., Martin N.G., Montgomery G.W., Whitfield J.B. (2009). Variants in TF and HFE explain approximately 40% of genetic variation in serum-transferrin levels. Am. J. Hum. Genet..

[38] Soranzo N., Spector T.D., Mangino M., Kühnel B., Rendon A., Teumer A., Willenborg C., Wright B., Chen L., Li M. (2009). A genome-wide meta-analysis identifies 22 loci associated with eight hematological parameters in the HaemGen consortium. Nat. Genet..

[39] Ganesh S.K., Zakai N.A., van Rooij F.J., Soranzo N., Smith A.V., Nalls M.A., Chen M.H., Kottgen A., Glazer N.L., Dehghan A. (2009). Multiple loci influence erythrocyte phenotypes in the CHARGE Consortium. Nat. Genet..

[40] Hentze M.W., Muckenthaler M.U., Galy B., Camaschella C. (2010). Two to tango: Regulation of Mammalian iron metabolism. Cell.

[41] Core A.B., Canali S., Babitt J.L. (2014). Hemojuvelin and bone morphogenetic protein (BMP) signaling in iron homeostasis. Front. Pharmacol..

[42] Ganz T. (2013). Systemic iron homeostasis. Physiol. Rev..

[43] Kang C., Li Ji L. (2012). Role of PGC-1alpha signaling in skeletal muscle health and disease. Ann. N. Y. Acad. Sci..

[44] Handschin C., Chin S., Li P., Liu F., Maratos-Flier E., Lebrasseur N.K., Yan Z., Spiegelman B.M. (2007). Skeletal muscle fiber-type switching, exercise intolerance, and myopathy in PGC-1alpha muscle-specific knock-out animals. J. Biol. Chem..

[45] Lin J., Wu H., Tarr P.T., Zhang C.Y., Wu Z., Boss O., Michael L.F., Puigserver P., Isotani E., Olson E.N. (2002). Transcriptional co-activator PGC-1 alpha drives the formation of slow-twitch muscle fibres. Nature.

[46] Yvert T., Miyamoto-Mikami E., Tobina T., Shiose K., Kakigi R., Tsuzuki T., Takaragawa M., Ichinoseki-Sekine N., Perez M., Kobayashi H. (2020). PPARGC1A rs8192678 and NRF1 rs6949152 Polymorphisms Are Associated with Muscle Fiber Composition in Women. Genes.

[47] Merrill J.F., Thomson D.M., Hardman S.E., Hepworth S.D., Willie S., Hancock C.R. (2012). Iron deficiency causes a shift in AMP-activated protein kinase (AMPK) subunit composition in rat skeletal muscle. Nutr. Metab..

[48] Willis W.T., Brooks G.A., Henderson S.A., Dallman P.R. (1987). Effects of iron deficiency and training on mitochondrial enzymes in skeletal muscle. J. Appl. Physiol..

[49] Rensvold J.W., Ong S.E., Jeevananthan A., Carr S.A., Mootha V.K., Pagliarini D.J. (2013). Complementary RNA and protein profiling identifies iron as a key regulator of mitochondrial biogenesis. Cell Rep..

[50] Zong H., Ren J.M., Young L.H., Pypaert M., Mu J., Birnbaum M.J., Shulman G.I. (2002). AMP kinase is required for mitochondrial biogenesis in skeletal muscle in response to chronic energy deprivation. Proc. Natl. Acad. Sci. USA.

[51] Wilson J.M., Loenneke J.P., Jo E., Wilson G.J., Zourdos M.C., Kim J.S. (2012). The effects of endurance, strength, and power training on muscle fiber type shifting. J. Strength Cond. Res..

[52] Harridge S.D. (2007). Plasticity of human skeletal muscle: Gene expression to in vivo function. Exp. Physiol..

[53] Jones S.W., Baker D.J., Gardiner S.M., Bennett T., Timmons J.A., Greenhaff P.L. (2004). The effect of the beta2-adrenoceptor agonist prodrug BRL-47672 on cardiovascular function, skeletal muscle myosin heavy chain, and MyoD expression in the rat. J. Pharmacol. Exp. Ther..

