# Supplementary material for: Genotype Score for Iron Status Is Associated with Muscle Fiber Composition in Women
Source: Genes (Basel). 2021 Dec 21;13(1):5. doi: 10.3390/genes13010005 (PMC8775127; doi:10.3390/genes13010005)
Supplement: Supplementary file 1 [file genes-13-00005-s001.zip › genes-1513519-supplementary.pdf]

Supplemental Table S1. Proportions of MHC-I, MHC-IIa, and MHC-IIx isoforms by genotypes in the *TMPRSS6* rs855791 T/C and the *HFE* rs1799945 C/G polymorphisms for all participants, men, and women.

Gene name (rs number)	Genotype		
All			
TMPRSS6 (rs855791)	TT (n = 68)	TC (n = 105)	CC (n = 41)
MHC-I (%)	47.9 ± 11.8	43.8 ± 11.9	46.0 ± 13.8
MHC-IIa (%)	31.6 ± 8.6	33.9 ± 8.2	35.0 ± 9.2
MHC-IIx (%)	20.5 ± 7.8	22.4 ± 8.9	19.0 ± 10.6
HFE (rs1799945)	CC (n = 203)	CG (n = 11)	GG (n = 0)
MHC-I (%)	45.4 ± 12.4	48.0 ± 11.2	
MHC-IIa (%)	33.5 ± 8.7	30.1 ± 6.4	
MHC-IIx (%)	21.1 ± 8.9	21.8 ± 10.1	
Men			
TMPRSS6 (rs855791)	TT (n = 33)	TC (n = 54)	CC (n = 20)
MHC-I (%)	44.3 ± 11.6	39.3 ± 10.6	38.8 ± 13.0
MHC-IIa (%)	35.3 ± 8.3	35.7 ± 8.0	37.3 ± 8.9
MHC-IIx (%)	20.4 ± 7.7	25.1 ± 9.0	23.9 ± 10.3
HFE (rs1799945)	CC (n = 100)	CG (n = 7)	GG (n = 0)
MHC-I (%)	40.7 ± 11.8	41.6 ± 11.8	
MHC-IIa (%)	36.2 ± 8.4	31.4 ± 3.8	
MHC-IIx (%)	23.2 ± 9.1	27.0 ± 7.4	
Women			
TMPRSS6 (rs855791)	TT (n = 35)	TC (n = 51)	CC (n = 21)
MHC-I (%)	51.4 ± 11.0	48.5 ± 11.3	52.9 ± 11.0
MHC-IIa (%)	28.2 ± 7.4	32.0 ± 8.0	32.7 ± 9.1
MHC-IIx (%)	20.5 ± 8.0	19.5 ± 7.9	14.4 ± 8.7
HFE (rs1799945)	CC (n = 103)	CG (n = 4)	GG (n = 0)
MHC-I (%)	50.0 ± 11.2	59.3 ± 4.9	
MHC-IIa (%)	31.0 ± 8.2	27.9 ± 9.9	
MHC-IIx (%)	19.0 ± 8.3	12.8 ± 7.6	

Data are expressed as means ± standard deviation, MHC: Myosin heavy chain
